# MAFLD with central obesity is associated with increased risk of colorectal adenoma and high-risk adenoma

**DOI:** 10.1186/s12876-024-03220-z

**Published:** 2024-04-22

**Authors:** Jingfang Xiong, Yijun Wu, Dongya Chen, Zhaolin Zhang, Yihui Liu, Jiandong Luo, Hong Xu

**Affiliations:** 1 Department of Geriatrics, Hangzhou Red Cross Hospital, Hangzhou City, Zhejiang Province China; 2 The 2nd Clinical Medical College, Zhejiang Chinese Medical University, Hangzhou City, Zhejiang Province China; 3 Department of Gastroenterology and Hepatology, Hangzhou Red Cross Hospital, Hangzhou City, Zhejiang Province China; 4 Endoscopy Center, Hangzhou Red Cross Hospital, Hangzhou City, Zhejiang Province China; 5 Department of Gastroenterology and Hepatology, Hangzhou Red Cross Hospital, 208 Huancheng Dong Road, 310003 Hangzhou, China

**Keywords:** Colorectal adenoma, MAFLD, Central obesity, High-risk adenoma

## Abstract

**Objective:**

To analyze the risk factors associated with colorectal adenoma and to investigate the associations of metabolism-related fatty liver disease (MAFLD) with obesity, colorectal adenoma and high-risk adenoma.

**Methods:**

A total of 1395 subjects were enrolled and divided into a colorectal adenoma group (593 subjects) and a control group (802 subjects) according to the inclusion and exclusion criteria. The characteristics of patients in the colorectal adenoma group and the control group were compared by the chi-square test. Univariate and multivariate logistic analyses were used to analyze independent risk factors and associations with different MAFLD subtypes. Colorectal adenoma characteristics and the proportion of patients with high-risk colorectal adenoma were also compared.

**Results:**

High-density lipoprotein (HDL-C) was significantly lower in patients in the colorectal adenoma group than in those in the control group (*P* < 0.001). Logistic regression analysis revealed that age, obesity status, central obesity status, hypertension status, diabetes status, fatty liver status, smoking history, BMI, waist circumference, triglyceride level, HDL-C level, fasting blood glucose level and degree of hepatic steatosis were all independent risk factors for colorectal adenoma. Notably, MAFLD was associated with a significantly increased risk of colorectal adenoma in patients with central obesity (*P* < 0.001). In addition, obesity, central obesity, diabetes, fatty liver and degree of hepatic steatosis were all shown to be independent risk factors for high-risk colorectal adenoma. In addition, a greater proportion of MAFLD patients with central obesity than those without central obesity had high-risk colorectal adenoma.

**Conclusion:**

MAFLD and central obesity are independently associated with the development of colorectal adenoma. MAFLD with central obesity is associated with an increased risk of colorectal adenoma and high-risk adenoma.

## Introduction

Colorectal carcinoma (CRC) is a common malignant tumor of the digestive system. According to the latest statistics in 2021, the number of new CRC cases worldwide has risen to 1.93 million, while the number of CRC-related deaths is nearly 940,000, ranking third and second in the global cancer ranking, respectively [1]. CRC is difficult to treat and has a poor prognosis, and both surgical treatment and chemoradiotherapy can have a substantial negative impact on the physical, psychological, and economic aspects of patients [2]. The adenoma-adenocarcinoma pathway is the most important route of CRC in humans [3]. Approximately 90% of CRC progresses from colorectal adenomas [4]. Approximately 5% of colorectal adenomas can progress from malignant to CRC, and its progression takes approximately 5–10 years [5]. Therefore, early prevention of colorectal adenoma, a precancerous lesion of CRC, is extremely critical.

As early as 2010, the Korean scholars Hwang et al. first demonstrated that nonalcoholic fatty liver disease (NAFLD) was associated with adenomatous polyps of the colon [6]. In recent years, many studies have shown that NAFLD is an independent risk factor for the occurrence of colorectal adenoma. A greater degree of hepatic steatosis in NAFLD patients was associated with a greater risk of colorectal adenoma [7, 8]. In addition, a large number of studies have shown that the incidence of colorectal adenoma is closely related to dietary pattern, lifestyle, metabolic syndrome, obesity, hypertension, lipid disorders and T2DM [9, 10]. NAFLD is considered to be a manifestation of metabolic syndrome in the liver and has many common risk factors for the occurrence and development of adenomatous polyps, such as obesity, diabetes, and metabolic syndrome. It is speculated that the risk of colorectal adenoma polyps in NAFLD patients may increase due to the increase in the above risk factors [11]. The term metabolism-related fatty liver disease (MAFLD) was renamed NAFLD. In March 2020, the International Panel on Fatty Liver Disease published a consensus statement on the new definition of MAFLD in the Journal of Hepatology [12]. The new diagnostic criteria for MAFLD are based on histological, imaging, and/or blood biomarker tests of liver biopsies suggesting the presence of fatty liver while meeting one of the following three conditions: overweight/obesity, type 2 diabetes mellitus (T2DM), or metabolic dysfunction [12, 13].

Previous studies have shown that nonobese MAFLD patients have a greater risk of developing colorectal adenoma, but the sample size was small, with only 124 patients [14]. In addition, although a large body of data suggests that MAFLD and obesity are risk factors for colorectal polyps, some scholars believe that the diagnosis of MAFLD is not sufficient to recommend colonoscopy [15–17]. There are currently many studies on colorectal adenoma, but the results are variable and controversial. Whether MAFLD patients and obese patients are at high risk for developing colorectal polyps remains unclear. MAFLD and colorectal polyps have common risk factors, and it is necessary to conduct studies with larger sample sizes and include more influencing factors to explore the correlation between the two through further in-depth study. In addition, we clinically observed that patients with MAFLD accompanied by obesity, particularly central obesity, had a greater risk of developing colorectal adenoma.

The present study is the first to report the characteristics of colorectal adenoma in Chinese patients with MAFLD and obesity. In addition, this study was designed to explore the potential relationships between MAFLD and obesity and between MAFLD and colorectal adenomas and high-risk adenomas from several perspectives. Determining the relationship between MAFLD combined with obesity and colorectal adenoma can provide a more robust clinical basis for early screening of colorectal adenoma-susceptible populations.

## Patients and methods

### Patients

A total of 1519 consecutive patients admitted to the Endoscopy Center, Hangzhou Red Cross Hospital between September 2022 and March 2023 were included in this study according to the following criteria.

The inclusion criteria were as follows: (1) complete medical history and clinical examination data; (2) complete colonoscopy, polypectomy and final pathology results; and (3) age between 20 and 80 years.

The exclusion criteria were as follows: (1) prior colorectal polypectomy; (2) a history of colorectal carcinoma or colorectal surgery; (3) inflammatory bowel disease (Crohn’s disease, ulcerative colitis, etc.); (4) intestinal tuberculosis; (5) viral hepatitis; (6) autoimmune hepatitis or other liver diseases; and (7) excess alcohol consumption.

Finally, a total of 1395 patients, including 593 patients with colorectal adenomas and 802 control participants, were enrolled after exclusion from the present study.

### Basic information collection

All patients completed a detailed questionnaire on comorbidities (hypertension, diabetes, hyperlipidemia, coronary heart disease), alcohol intake, smoking status, medication use (antihypertensive, hypoglycemic, or hypolipidemic drugs) and colorectal cancer in first-degree relatives. Body weight (kg) and height (m) were measured, and body mass index (BMI) was calculated as weight/height^2^ (kg/m^2^). Obesity was defined as a BMI ≥ 25 kg/m^2^ [18]. Resting blood pressure was measured using a corrected electronic sphygmomanometer. Waist circumference was measured by a well-trained person using a tape measure at the midpoint between the lower costal margin and the anterior superior iliac crest. Hypertension, T2DM, and dyslipidemia were diagnosed according to standard criteria [12, 19, 20].

### Biochemical analysis

The subjects fasted overnight prior to blood sampling. Triglyceride, high-density lipoprotein (HDL) cholesterol and glucose were measured by an automatic biochemical analyzer (Beckman Coulter AU 5800).

### Colonoscopic examination

Bowel preparation was performed the night before colonoscopy. Bowel preparation criteria were as follows: 4–6 watery stools should be discharged after drinking laxatives, and the Boston score should be greater than 7 points. All the subjects were examined by experienced endoscopists who had performed more than 5000 procedures with video colonoscopy (Olympus PCF-H290). A complete colonoscopic examination was defined as an endoscope reaching the cecum as documented by a picture of the ileocecal valve. The number, size, location and shape of the polyps were carefully observed and recorded. Adenoma size was assessed using open colonoscopic biopsy forceps and classified as < 10 or ≥ 10 mm. The largest size was recorded for multiple adenomas. Colorectal polyps were located in the proximal colon (cecum, ascending colon, and transverse colon), distal colon (splenic flexure, descending colon, sigmoid colon, and rectum), and both sides of the colon. According to the size and shape of the polyps, biopsy forceps, cold snare polypectomy (CSP), hot snare polypectomy (HSP) or endoscopic mucosal resection (EMR) were used.

### Pathology

The removed samples were fixed and used for histological analysis. The colorectal adenomas included tubular, villous, or serrated adenomas. In patients with multiple lesions, the most advanced pathology was selected as the definitive lesion. According to the updated United States Multi-Society Task Force (USMSTF) guidelines, high-risk adenoma is defined as an advanced adenoma (villous adenoma, high-grade dysplasia, or ≥ 10 mm) and/or the presence of 3 or more adenomas [21]. Control participants were characterized by normal colonoscopic findings and nonpolypoid benign lesions such as nonspecific colitis or histologically confirmed hyperplastic polyps.

### Diagnosis of MAFLD

First, the diagnosis of MAFLD requires abdominal imaging to confirm the presence of fatty liver. In brief, all patients underwent abdominal ultrasonography (Philips iu22 color ultrasonic diagnostic apparatus) after fasting for at least 8 h. Fatty liver was diagnosed based on characteristic ultrasonographic findings of increased hepatorenal contrast, brightness of the liver parenchyma, and impaired visualization of the diaphragm or intrahepatic vessels [22]. Using ultrasonographic findings to diagnose hepatic steatosis and a standardized algorithm, we classified the degree of steatosis into 4 categories (normal, mild, moderate, or severe).

The diagnostic criteria for MAFLD are based on abdominal imaging examination suggesting the presence of fatty liver while meeting one of the following three conditions: overweight/obesity, T2DM, or nonobesity with metabolic dysregulation. Metabolic dysregulation was defined as the presence of at least two metabolic risk abnormalities: waist circumference ≥ 90/80 cm for men/women; blood pressure ≥ 130/85 mmHg or receiving antihypertensive therapy; plasma triglycerides ≥ 150 mg/dL or receiving antihypertriglyceridemic therapy; and plasma HDL-cholesterol < 40/50 mg/dL for men/women or receiving antihypercholesterolemic therapy [12].

### Statistical analysis

Statistical Package for the Social Sciences (SPSS version 27.0) was used for statistical analysis. Continuous variables are expressed as the mean ± standard deviation (for normally distributed variables) or median values (interquartile ranges) (for nonnormally distributed variables). Student’s *t* test or the Mann‒Whitney U test was applied as appropriate to compare continuous variables. Categorical variables are expressed as percentages and were analyzed by the chi-square test. Univariate and multivariate analyses (logistic regression) were used to analyze the risk factors for colorectal adenoma. A *P* value less than 0.05 was considered to indicate statistical significance.

## Results

### General clinical data

According to the inclusion and exclusion criteria, a total of 1395 patients were enrolled in this study. Among these, 593 patients with colorectal adenomas and 802 control participants were included in the final analysis. The clinical and biochemical characteristics of the patients in the colorectal adenoma and control groups are shown in Table 1. The sex distribution was not significantly different between colorectal adenoma patients and control participants (*P* > 0.05), while the age distribution was significantly different (*P* < 0.001). In addition, BMI, waist circumference, serum triglycerides, and fasting blood glucose were significantly greater (all *P* < 0.001), while serum HDL-C was significantly lower (*P* < 0.001) in patients with colorectal adenomas than in control participants. Hypertension was diagnosed in 200 (33.7%), diabetes mellitus in 141 (23.8%), obesity in 381 (64.2%), central obesity in 168 (28.3%), and fatty liver in 341 (57.5%) patients with colorectal adenomas. Compared with those in the control group, the proportions of patients with hypertension, diabetes, obesity and central obesity were significantly greater in the colorectal adenoma group (all *P* < 0.001), suggesting that the occurrence of colorectal adenoma was significantly associated with the above diseases. Notably, abdominal ultrasonography revealed a greater proportion of patients with fatty liver in the colorectal adenoma group (341 patients, 57.5%) than in the control group, which suggested that the occurrence of colorectal adenoma is also significantly associated with the development of fatty liver tissue.

**Table 1:** Patient clinical data

Characteristic	Colorectal adenoma (*n* = 593)	Control (*n* = 802)	P
Sex (Male/Female)	376/217	489/313	0.355
Age	57 (46, 66)	52 (44, 60)	< 0.001
BMI	25.7 (24.6, 26.8)	24.5 (23.5, 25.5)	< 0.001
Waist circumference (cm)	85.6 (80.9, 90.5)	80.8 (77.0, 86.1)	< 0.001
Triglyceride (mg/dL)	126.0 (105.2, 143.1)	98.7 (75.8, 119.6)	< 0.001
HDL-cholesterol (mg/dL)	46.6 (40.6, 51.7)	56.3 (52.1, 60.4)	< 0.001
Fasting glucose (mg/dL)	100.0 (94.4, 105.0)	95.8 (90.0, 101.0)	< 0.001
Hypertension (%)	200 (33.7%)	137 (17.1%)	< 0.001
Diabetes	141 (23.8%)	41 (5.1%)	< 0.001
Obesity	381 (64.2%)	300 (37.4%)	< 0.001
Central obesity	168 (28.3%)	47 (5.9%)	< 0.001
Smoking	124 (20.9%)	95 (11.8%)	< 0.001
Fatty liver	341 (57.5%)	286 (35.6%)	0.005

The Z test was used to compare proportions of two independent samples. The Mann‒Whitney U test was used to compare nonnormally distributed continuous variables

### Clinical features of colonic adenomatous polyps

Of the 1395 enrolled patients, 593 (42.5%) had colorectal adenomas. The characteristics of the 593 patients with colorectal adenomas are shown in Table 2. The majority of patients with adenomas (*n* = 557; 93.9%) had < 3 adenomas. Adenomas were more frequently distributed on the proximal end (341 patients, 57.5%) than on the distal end (129 patients, 21.8%) or both ends (123 patients, 20.7%) of the colon. Most adenomas (552 patients, 93.1%) were less than 10 mm in size. Pathologically, 50 (8.4%) of the patients had advanced adenomatous lesions. According to the diagnostic criteria for high-risk adenoma, 66 patients (11.1%) were identified as having high-risk adenoma among the 593 patients with colorectal adenoma.

**Table 2:** Clinical characteristics of colorectal adenomas in patients

Characteristic		Number of subjects (N/%)
Number of colorectal adenoma	< 3	532 (89.7%)
	≥ 3	61 (10.3%)
Location	Proximal	341 (57.5%)
	Distal	129 (21.8%)
	Both	123 (20.7%)
Size (mm)	< 10	552 (93.1%)
	≥ 10	41 (6.9%)
Advanced adenoma	Yes	50 (8.4%)
	No	543 (91.6%)
High-risk adenoma	Yes	66 (11.1%)
	No	527 (88.9%)

### Univariate and multivariate analyses of the predictive factors associated with colorectal adenoma

We investigated the associations between colorectal adenomas and different risk factors (Table 3). Multiple regression analyses were performed using stepwise procedures, which included all confounding factors as independent variables. Univariate logistic analysis revealed that age, obesity status, central obesity status, hypertension status, diabetes status, fatty liver status and smoking history were significantly associated with the occurrence of colorectal adenoma (all *P* < 0.01). The calculated ORs all showed a significantly increased risk of colorectal adenoma in the following individuals: those aged ≥ 60 years (1.25-fold), those with obesity (2.01-fold), those with central obesity (5.35-fold), those with hypertension (1.47-fold), those with diabetes (4.79-fold), those with fatty liver (1.36-fold), and those who smoked (0.97-fold). Among the continuous variables, changes in BMI, waist circumference, triglyceride levels, HDL-C levels, and fasting glucose levels were all significantly associated with the development of colorectal adenomas (all *P* < 0.001). The ORs showed a significant 1.14-, 0.05-, 0.05-, and 0.11-fold increased risk of colorectal adenoma with increasing BMI, waist circumference, triglyceride level, or fasting glucose, respectively. In addition, there was a significant 0.24-fold reduction in the risk of colorectal adenoma with increasing HDL-C levels. Notably, the risk associated with central obesity, diabetes and fatty liver significantly increased by 2.27-, 1.17- and 0.94-fold, respectively, after adjustment for these factors.

**Table 3:** Logistic regression analysis of the risk of colorectal adenoma

Characteristic		Colorectal adenoma (*n* = 593)	Control (*n* = 802)	Univariate analysis		Multivariate analysis	
				OR (95%CI)	P	OR (95%CI)	P
Age	< 60	326	588	2.3 (1.8, 2.8)	< 0.001	2.2 (1.4, 3.4)	< 0.001
	≥ 60	267	214				
Sex	Male	376	489	0.9 (0.7, 1.1)	0.355	0.9 (0.6, 1.5)	0.778
	Female	217	313				
Obesity	Yes	381	300	3.0 (2.4, 3.8)	< 0.001	0.1 (0.1, 0.3)	< 0.001
	No	212	502				
Central obesity	Yes	168	47	6.4 (4.5, 9.0)	< 0.001	3.3 (1.7, 6.2)	< 0.001
	No	425	755				
Hypertension	Yes	200	137	2.5 (1.9, 3.2)	< 0.001	2.7 (1.8, 4.2)	< 0.001
	No	393	665				
Diabetes	Yes	141	41	5.8 (4.0, 8.4)	< 0.001	2.2 (1.1, 4.1)	0.018
	No	452	761				
Fatty liver	Yes	341	286	2.4 (1.1, 3.7)	0.005	1.9 (0.6, 3.4)	0.007
	No	252	536				
Smoking	Yes	124	95	2.0 (1.5, 2.6)	< 0.001	2.6 (1.5, 4.4)	< 0.001
	No	469	707				
BMI		25.7 (24.6, 26.8)	24.5 (23.5, 25.5)	2.1 (1.9, 2.4)	< 0.001	4.3 (3.1, 5.9)	< 0.001
Waist circumference (cm)		85.6 (80.9, 90.5)	80.8 (77.0, 86.1)	1.1 (1.0, 1.1)	< 0.001	1.0 (1.0, 1.0)	0.846
Triglyceride (mg/dL)		126.0 (105.2, 143.1)	98.7 (75.8, 119.6)	1.1 (1.0, 1.1)	< 0.001	1.1 (1.0, 1.1)	< 0.001
HDL-cholesterol (mg/dL)		46.6 (40.6, 51.7)	56.3 (52.1, 60.4)	0.8 (0.7, 0.8)	< 0.001	0.8 (0.7, 0.8)	< 0.001
Fasting glucose (mg/dL)		100.0 (94.4, 105.0)	95.8 (90.0, 101.0)	1.1 (1.1, 1.1)	< 0.001	1.1 (1.1, 1.1)	< 0.001

Given that diabetes and obesity are major risk factors contributing to the development of colorectal adenomas, we further explored the associations of MAFLD with central obesity or diabetes and the risk of colorectal adenoma development. As shown in Table 4, there was a high proportion of colorectal adenomas in patients with MAFLD accompanied by central obesity or diabetes (81.5% [97/119] and 82.1% [78/95], respectively). In addition, compared with patients without fatty liver, lean MAFLD patients and MAFLD patients with obesity, central obesity, or diabetes had a significantly increased risk of developing colorectal adenomas (all *P* < 0.001). Interestingly, compared with a 1.28-fold increase in the MAFLD with obesity group, patients in the MAFLD with central obesity group had a 6.00-fold increased risk of colorectal adenoma, suggesting that central obesity is more likely to lead to colorectal adenoma development. Notably, patients with non-MAFLD fatty liver had a significantly decreased risk of colorectal adenoma development (*P* < 0.001).

**Table 4:** Logistic regression analysis of colorectal adenoma risk and different MAFLD types

Different types	Colorectal adenoma	Control	OR (95%CI)	P
No fatty liver	252	536	1	
Non-MAFLD fatty liver	47	190	0.4 (0.3, 0.6)	< 0.001
MAFLD (Lean)	17	23	2.3 (1.5, 3.6)	< 0.001
MAFLD (Obesity)	215	150	2.3 (1.8, 3.0)	< 0.001
MAFLD (Central obesity)	97	22	7.0 (4.3, 11.4)	< 0.001
MAFLD (Diabetes)	78	17	7.3 (4.2, 12.6)	< 0.001

### Correlation analysis of different degrees of steatosis with colorectal adenoma and high-risk colorectal adenoma

Given that fatty liver is an independent risk factor for colorectal adenoma, we further analyzed the association between different degrees of steatosis and colorectal adenoma. As shown in Table 5, the proportion of patients who developed colorectal adenomas gradually increased as the degree of steatosis increased. The risk of colorectal adenoma was significantly increased by 0.95-fold and 6.74-fold in patients with severe and moderate steatosis, respectively, suggesting that severe steatosis and moderate steatosis are independent risk factors for the development of colorectal adenoma. Interestingly, the risk of high-risk colorectal adenomas was significantly increased by 1.76-, 4.68-, and 9.42-fold in patients with mild, moderate, and severe steatosis, respectively (Table 6), suggesting that varying degrees of steatosis are independent risk factors for the development of high-risk colorectal adenomas.

**Table 5:** Logistic regression analysis of colorectal adenoma risk and different degrees of steatosis

Different types	Colorectal adenoma (*n* = 593)	Control (*n* = 802)	OR (95%CI)	P
Normal	262	416	1	
Mild steatosis	222	321	1.1 (0.9, 1.4)	0.426
Moderate steatosis	70	57	2.0 (1.3, 2.9)	< 0.001
Severe steatosis	39	8	7.7 (3.6, 16.8)	< 0.001

**Table 6:** Logistic regression analysis of high-risk colorectal adenoma risk and different degrees of steatosis

Different types	High-risk adenoma (*n* = 66)	Low-risk adenoma (*n* = 527)	OR (95%CI)	P
Normal	12	250	1	
Mild steatosis	26	196	2.8 (1.4, 5.6)	0.005
Moderate steatosis	15	55	5.7 (2.5, 12.8)	< 0.001
Severe steatosis	13	26	10.4 (4.3, 25.2)	< 0.001

### Univariate and multivariate analysis of the predictive factors associated with high-risk adenomas

Subsequently, we analyzed the associations between high-risk adenomas and different risk factors in patients with colorectal adenomas (Table 7). Multiple regression analyses were performed using stepwise procedures, including all confounding factors as independent variables. Univariate logistic analysis revealed that the presence of obesity, central obesity, diabetes, and fatty liver was significantly associated with the occurrence of high-risk colorectal adenomas (all *P* < 0.001). The calculated ORs showed a significant 20.20-, 10.92-, 1.00-, and 3.06-fold increased risk of high-risk colorectal adenomas in individuals with obesity, central obesity, diabetes, or fatty liver, respectively. Among the continuous variables, increases in BMI and waist circumference were significantly associated with the development of high-risk colorectal adenomas (*P* < 0.001 and 0.05, respectively). The calculated ORs further showed a significant 0.84-fold and 0.07-fold increased risk of high-risk colorectal adenomas with increasing BMI and waist circumference, respectively. Notably, central obesity and fatty liver status remained significantly associated with the development of high-risk colorectal adenomas after adjusting for other factors (all *P* < 0.01).

**Table 7:** Logistic regression analysis of the risk of high-risk colorectal adenoma

Characteristic		High-risk adenoma (*n* = 66)	Low-risk adenoma (*n* = 527)	Univariate analysis	Multivariate analysis		
				OR (95%CI)	P	OR (95%CI)	P
Age	< 60	26	300	2.0 (1.2, 3.4)	0.008	1.1 (0.6, 2.0)	0.878
	≥ 60	40	227				
Sex	Male	27	349	2.8 (1.7, 4.8)	< 0.001	1.1 (0.5, 2.5)	0.738
	Female	39	178				
Obesity	Yes	64	317	21.2 (5.1, 87.5)	< 0.001	5.5 (1.0, 31.2)	0.054
	No	2	210				
Central obesity	Yes	51	117	11.9 (6.5, 22.0)	< 0.001	12.7 (4.2, 38.8)	< 0.001
	No	15	410				
Hypertension	Yes	21	179	0.9 (0.5, 1.6)	0.728	1.2 (0.6, 2.3)	0.559
	No	45	348				
Diabetes	Yes	24	117	2.0 (1.2, 3.4)	0.012	2.0 (0.9, 4.1)	0.079
	No	42	410				
Fatty liver	Yes	54	277	4.1 (2.1, 7.8)	< 0.001	5.7 (2.7, 11.8)	< 0.001
	No	12	250				
Smoking	Yes	14	110	1.0 (0.6, 1.9)	0.949	1.1 (0.5, 2.3)	0.783
	No	52	417				
BMI		26.5 (25.9, 27.1)	25.5 (24.4, 26.7)	1.8 (1.5, 2.3)	< 0.001	1.0 (0.7, 1.5)	0.997
Waist circumference (cm)		88.8 (82.6, 91.6)	85.3 (80.6, 90.1)	1.1 (1.0, 1.1)	0.013	0.9 (0.9, 1.0)	0.132
Triglyceride (mg/dL)		125.1 (102.2, 143.68)	126.2 (105.3, 142.7)	1.0 (1.0, 1.0)	0.611	1.0 (1.0, 1.0)	0.101
HDL-cholesterol (mg/dL)		46.8 (40.1, 51.7)	46.5 (40.7, 51.8)	1.0 (1.0, 1.1)	0.902	1.0 (1.0, 1.0)	0.789
Fasting glucose (mg/dL)		102.2 (95.8, 106.3)	99.6 (94.2, 104.9)	1.0 (1.0, 1.1)	0.070	1.0 (1.0, 1.1)	0.547

### Characteristics of colorectal adenomas in MAFLD patients with concomitant obesity

Finally, we compared the characteristics of colorectal adenomas between MAFLD patients with or without central obesity. As shown in Table 8, more patients in the MAFLD with central obesity group had colorectal adenomas *(P* < 0.001). Among patients with colorectal adenomas, the proportions of patients with adenomas distributed distally (25.8%) or at both ends (34.0%) were significantly greater in the MAFLD with central obesity group than in the noncentral obesity MAFLD group (distal: 21.0%; both: 18.1%) (both *P* < 0.001). In addition, the proportion of adenomas ≥ 10 mm in size and the frequency of advanced adenomas was significantly greater in MAFLD patients with central obesity than in MAFLD patients without central obesity (all *P* < 0.001). In addition, the proportion of high-risk adenomas was significantly greater in the centrally obese MAFLD group than in the noncentrally obese MAFLD group (42.3% vs. 5.0%, *P* < 0.001).

**Table 8:** Colorectal Adenoma Characteristics in Patients with MAFLD with and without Central Obesity

Characteristic		MAFLD with central obesity (*n* = 97)	MAFLD without central obesity (*n* = 496)	OR (95%CI)	P
Numbers	< 3	60	472	12.1 (6.8, 21.7)	< 0.001
	≥ 3	37	24		
Location	Proximal	39	302	/	< 0.001
	Distal	25	104		
	Both	33	90		
Size (mm)	< 10	73	479	9.3 (4.7, 18.1)	< 0.001
	≥ 10	24	17		
Advanced adenoma	Yes	29	21	9.6 (5.2, 17.9)	< 0.001
	No	68	475		
High-risk adenoma	Yes	41	25	13.8 (7.8, 24.4)	< 0.001
	No	56	471		

## Discussion

MAFLD is a multisystem disease associated with metabolic disorders and is closely related to the high incidence of metabolic syndrome, T2DM, cardiovascular disease, cirrhosis, hepatocellular carcinoma, colorectal adenomatous polyps and other diseases [18]. The diagnostic criteria following the renaming of MAFLD highlight the important role of metabolic dysfunctions in the development of fatty liver disease and may be more appropriate for identifying patients with fatty liver disease at high risk of disease progression [13]. CRC is a malignant tumor with a high mortality rate, and CRC incidence is increasing annually; therefore, early prevention of CRC is particularly important [23]. A large number of studies and clinical practices have shown that colorectal cancer screening and early diagnosis and treatment can effectively reduce the incidence and mortality of colorectal cancer [24, 25]. Colorectal adenoma is a protuberant lesion protruding into the intestinal lumen originating from the colorectal epithelial layer that lacks specific symptoms, although a few patients may experience abdominal pain, diarrhea, constipation, hematochezia, and intestinal obstruction [25]. Colorectal adenoma, a precancerous lesion of CRC, has been gradually studied.

Studies have shown that obesity, T2DM and metabolic syndrome are closely related to colorectal adenoma and flat adenomas [16]. In addition, overweight or obesity is causally associated with an increased incidence of T2DM, cardiovascular disease, and several forms of malignancy [26]. MAFLD is a manifestation of metabolic syndrome in the liver, and studies have shown an increased risk of colorectal adenomas in patients with MAFLD [14]. Given the close association between MAFLD and colorectal adenomas, obesity can in turn be used as an indicator for the diagnosis of MAFLD. Therefore, whether there is a correlation between MAFLD combined with central obesity and colorectal adenoma deserves in-depth investigation.

Epidemiological studies have shown that the risk of colorectal polyps increases by 3% for every year increase in age [27]. A Korean study showed that metabolic syndrome, obesity, advanced age, and male sex were independent risk factors for colorectal adenoma [28]. In our study, patients in the colorectal adenoma group were significantly older than those in the control group (*P* < 0.001). In addition, univariate and multivariate logistic regression analyses revealed that having an age ≥ 60 years was a high risk factor for colorectal adenoma development. Several studies have suggested that obesity is a significant risk factor for the development of colorectal adenoma. Ashktorab et al. showed that overweight status was significantly associated with an increased risk of colorectal adenoma in African Americans [15]. In Europe, epidemiological data have shown that 11% of colorectal adenomas are attributable to overweight and obesity [29]. Chinese scholars screened and followed-up a total of 20,811 community residents as part of the colorectal cancer community screening program in Minhang District, Shanghai [30]. Their results showed that the risk of colorectal high-risk adenoma was significantly increased in people with obesity, and the risk was greater in people > 60 years old. In this study, we found that both BMI and the proportion of patients suffering from obesity and central obesity were significantly greater in the colorectal adenoma group than in the control group (*P* < 0.001). A comparison of the waist circumference between the two groups revealed that the waist circumference of the colorectal adenoma group was also greater than that of the control group, and the difference was statistically significant (*P* < 0.001). Univariate logistic regression analysis revealed that BMI, waist circumference, obesity and central obesity were all independent risk factors for colorectal adenoma. Interestingly, the risk of colorectal adenoma development was significantly increased 3.01-fold or 6.35-fold when obesity or central obesity was present, suggesting that central obesity is associated with a greater risk of colorectal adenoma development. However, in multivariate regression analysis, the difference in waist circumference was no longer statistically significant, which may have been due to enrollment bias. In patients with high-risk and low-risk colorectal adenomas, univariate logistic regression analysis revealed that BMI, waist circumference, obesity, and central obesity were all independent risk factors for the development of high-risk colorectal adenomas. Moreover, after adjusting for other factors, central obesity remained an independent risk factor for the development of high-risk colorectal adenomas, suggesting that central obesity is significantly associated with high-risk colorectal adenomas.

Although some studies have identified an association between colorectal adenoma and fatty liver, the present study incorporated a more in-depth method for demonstrating the association between colorectal adenoma and the degree of hepatic steatosis. There is strong evidence that visceral adiposity increases the risk of colorectal adenomas [31, 32]. In addition, the degree of hepatic steatosis represents the severity of fatty liver. The results of this study showed that moderate and severe hepatic steatosis were independent risk factors for colorectal adenoma development. Moreover, varying degrees of hepatic steatosis are independent risk factors for the development of high-risk colorectal adenomas. In other words, hepatic fat accumulation may play a role in adenoma formation. Lipolysis of visceral fat occurs later than that of subcutaneous fat and may be important in the progression from adenoma to carcinoma [33]. This further supports the greater association of hepatic steatosis with high-risk adenoma development.

Several studies have shown that blood pressure and blood lipids are closely related to the occurrence of colorectal adenomas [34, 35]. Kim BC et al. reported that elevated blood pressure and dyslipidemia were associated with an increased risk of colorectal adenoma [34]. Jung YS et al. showed that serum triglycerides and total bile solids were positively correlated with the occurrence of colorectal adenoma and were risk factors for colorectal adenoma [35]. In addition, Kort et al. reported a relative increase in the incidence of adenomas in diabetic patients compared to nondiabetic patients [36]. However, their study failed to find an association between hypertension and fasting glucose in patients with colorectal adenomas. In this study, we compared biochemical parameters between the colorectal adenoma group and the control group and found that the triglyceride level, fasting blood glucose level, and number of patients suffering from hypertension and diabetes in the colorectal adenoma group were greater than those in the control group, while the HDL-C level was significantly lower in the colorectal adenoma group than in the control group (all *P* < 0.001). Similarly, univariate and multivariate logistic regression analyses revealed that triglyceride levels, HDL-C levels, fasting blood glucose levels, hypertension, and diabetes were all risk factors for colorectal adenoma. However, no significant associations were found for triglycerides, HDL-C, fasting plasma glucose, or hypertension in high-risk colorectal adenoma patients. Interestingly, univariate logistic regression analysis revealed that diabetes was an independent risk factor for the development of high-risk colorectal adenomas.

A large number of studies have confirmed a close correlation between colorectal adenoma and MAFLD [37, 38]. Bhatt et al., in a retrospective analysis of liver transplant patients, reported that patients with MAFLD had a 2-fold increased risk of colorectal adenoma relative to patients with other end-stage liver diseases [37]. A meta-analysis of 6263 subjects revealed a significant association between MAFLD and colorectal adenomas, and this association was more pronounced in Asian populations [38]. In this study, we investigated the association between MAFLD and the risk of colorectal adenoma development. The results showed that MAFLD was significantly associated with the development of colorectal adenomas. Given that diabetes, obesity, and central obesity are major risk factors contributing to colorectal adenoma development, we explored the associations of MAFLD combined with diabetes, obesity, or central obesity with the risk of colorectal adenoma development. Current data collectively demonstrated that patients with MAFLD who had concomitant obesity or central obesity had a 1.28- or 6.00-fold significantly increased risk of colorectal adenoma, respectively. Similarly, Zhang S et al. reported that obesity, especially central obesity, increases the incidence of colorectal polyps and that central obesity is independently associated with the development of adenomatous polyps [39]. Moreover, increased BMI has been found to increase the risk of CRC [40].

In this study, we investigated the characteristics of colorectal adenomas in patients with MAFLD combined with central obesity. As shown in Table 6, compared with those in the other groups, subjects in the MAFLD with central obesity group had adenomas with larger diameters, a greater number of colorectal adenomas, and a greater proportion of advanced adenomas. Moreover, compared with those in the non-central obesity group, subjects in the MAFLD with central obesity group had a significantly higher proportion of high-risk adenomas (*P* < 0.001). According to our review of the literature, this is the first study to show that MAFLD is positively associated with obesity and high-risk adenomas. Compared to previous studies, our study involved a relatively large cohort, so the results are representative. In addition, we focused on high-risk adenomas with higher levels of malignancy, including multiple or large adenomas and advanced colorectal tumors. Overall, we found that obesity is an independent risk factor for colorectal adenoma as well as high-risk colorectal adenoma development. Moreover, MAFLD was also significantly associated with the development of colorectal adenomas. The risk of high-risk colorectal adenomas is greater when MAFLD coexists with obesity. Therefore, the screening of colorectal adenomas characterized by high-risk colorectal adenomas in MAFLD patients with obesity has guiding significance and clinical value.

This study is very novel. Colorectal adenoma is a precancerous lesion of CRC, and early prevention is essential. This study was the first to analyze and identify risk factors for colorectal adenoma in nearly 1400 Chinese patients and to investigate the associations of MAFLD with obesity, colorectal adenoma, and high-risk adenoma. These findings can provide a more robust clinical basis for early screening of colorectal adenoma-susceptible populations. This study also has several limitations. First, the subjects included in this study were all Asian, and the analysis results may be biased; however, they provide a clearer clinical basis for early screening of colorectal adenoma-susceptible populations in the Asian region. Second, the exposure and outcome measures of this study are cross-sectional, and the direction of causality is uncertain. Third, there were limited risk factors included in the analysis, such as dietary habits and physical activity, and it is not clear whether the effects of these factors on colorectal adenomas and high-risk adenomas can be completely ignored.

MAFLD is independently associated with the development of colorectal adenomas. Patients with MAFLD combined with central obesity have a greater risk of developing high-risk colorectal adenomas. MAFLD combined with central obesity can be used to initially screen high-risk patients with colorectal adenomas or high-risk adenomas and recommend targeted colonoscopy for high-risk patients, which can improve the screening efficiency of colonoscopy and optimize the allocation of medical resources. Early screening for colorectal adenomas and high-risk adenomas in high-risk populations, followed by early prediction, detection, and treatment of colorectal adenomatous polyps, is important to reduce the incidence and mortality of CRC.

## Data Availability

All the data generated or analyzed during this study are included in this published article.
